# A case of inflammatory-related urethral tumor in a child

**DOI:** 10.1016/j.eucr.2025.103156

**Published:** 2025-08-14

**Authors:** Hongjia He, Meng Gui, Kaisheng Li

**Affiliations:** Department of Minimally Invasive Urology, Shandong University Children's Hospital, Jinan, China

**Keywords:** Pediatrics, Urethral mass, Urinary difficulty, Acute urinary retention

## Abstract

A rare pediatric urethral mass presented with penile root pain, urinary stream narrowing, and acute urinary retention without trauma or infection history. Examination revealed abdominal distension and a tender penile base mass. Laboratory and imaging findings indicated inflammation and a cystic lesion. After catheterization and antibiotics, symptoms resolved completely, and no recurrence was noted. Although cystoscopic resection is commonly advised, this case highlights that conservative management may be effective in selected patients, potentially sparing children from invasive procedures.

## 1 Introduction

Compared to adults, the occurrence of urethral masses in children is extremely rare and often presents with unique histological features. Rhabdomyosarcoma is the most common malignant tumor of the lower urinary tract in children; however, the majority of lower urinary tract masses are benign lesions.^1^ These benign masses include fibroepithelial polyps, inflammatory myofibroblastic tumors, urothelial papillomas, neurofibromas, leiomyomas, and eosinophilic cystitis. Children with urethral masses often present with typical symptoms, such as dysuria, narrow urine stream, frequency, hematuria, and even urinary retention.^2^

In the assessment of such children, ultrasound remains the preferred imaging diagnostic method; however, it is difficult to accurately determine the nature of the urethral mass. Therefore, almost all children with urethral masses require pathological examination after cystoscopic resection for definitive diagnosis. Moreover, diseases such as fibroepithelial polyps, urothelial papillomas, eosinophilic cystitis, and even rhabdomyosarcoma can present with similar findings on ultrasound. To date, there have been no reports on inflammatory urethral masses.^3^ This report describes a child with elevated peripheral blood infection markers who presented with a urethral mass; a cystic mass was palpable at the root of the penis, and imaging showed a low-density focus within the urethra.

## 2 Case presentation

A 7-year-old male child presented to Shandong University Children's Hospital with difficulty urinating and urinary retention. He had experienced urethral pain at the root of the penis one week prior without any obvious cause, accompanied by urinary frequency and a narrowed urine stream. These symptoms had not alleviated over the past week, and one day prior, the pain worsened, along with the onset of urinary difficulty and progression to urinary retention. His urine color was normal, and there were no visible hematuria, chills, fever, abdominal pain, diarrhea, or nausea/vomiting. He denied any trauma or recent upper respiratory infections. The child had no significant medical history, no surgical history, and no known food or drug allergies, with no family history of relevant diseases. Physical examination revealed a generally stable mental state, good nutritional status, and all vital signs within normal range. The lower abdomen was notably distended, and a soft, tender, limited-mobility mass approximately 1.0 × 0.5 cm in size was palpable at the ventral root of the penis, there is no obvious redness on the surface of the skin, no significant increase in skin temperature, and no obvious wave sensation on palpation.No other abnormalities were found upon abdominal examination. The remaining physical examination showed no additional abnormalities. Due to the absence of urination for one day and marked abdominal distension, a urinary catheter was placed in the emergency department.

**Fig. 1:**
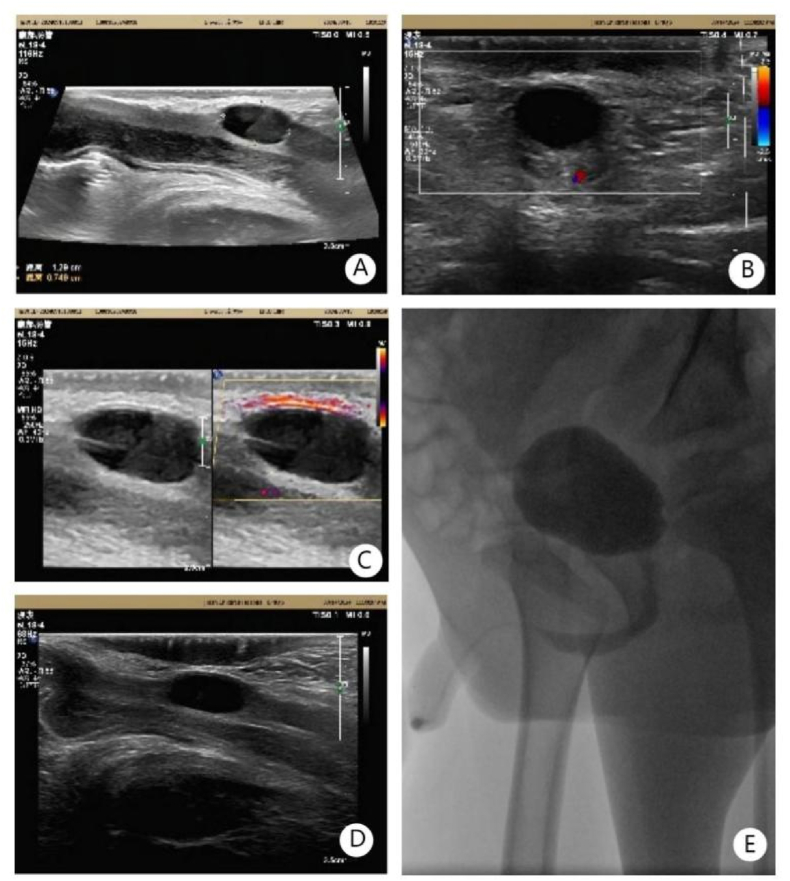
Ultrasound and retrograde cystography findings at admission. Panels A, B, C, and D show an oval-shaped cystic mass in the urethra observed on ultrasound, with clear margins, poor internal sound transmission, and visible layering; catheter echo is noted within the urethra. Panel E demonstrates retrograde cystography findings, indicating significant narrowing of the urethra anterior to the pubic bone, with slow passage of the contrast agent.

To identify the source and nature of the urethral mass, we conducted a series of laboratory and imaging examinations. Blood cell analysis revealed a white blood cell count of 15.24 × 10ˆ9/L, with a neutrophil percentage of 83.5 %, suggesting a possible infection. Urinalysis and other laboratory tests showed no abnormalities. Urethral ultrasound revealed an oval cystic mass at the posterior wall of the urethral root, measuring approximately 1.3 × 0.8 × 0.7 cm, with clear borders, poor sound transmission, and a layered appearance, along with dense punctate echoes(Fig. 1). Abdominal and pelvic CT scans showed a small cystic low-density lesion in the urethral region with poorly defined borders. The bladder was well distended, and there was no obvious localized thickening of the wall or abnormal low-density lesions in the cavity. No enlarged lymph nodes were noted in the pelvis, but a small amount of fluid-density lesions were seen in the pelvic cavity. Urethrography showed significant narrowing of the urethra anterior to the pubic bone, with slow contrast passage.

During hospitalization, a Foley catheter was placed, and the patient was treated with cefuroxime 40 mg/kg twice a day for infection control. After five days, a follow-up blood test showed normalization of white blood cell count, neutrophils, and C-reactive protein. A repeat urethral ultrasound revealed resolution of the urethral mass.

**Fig. 2:**
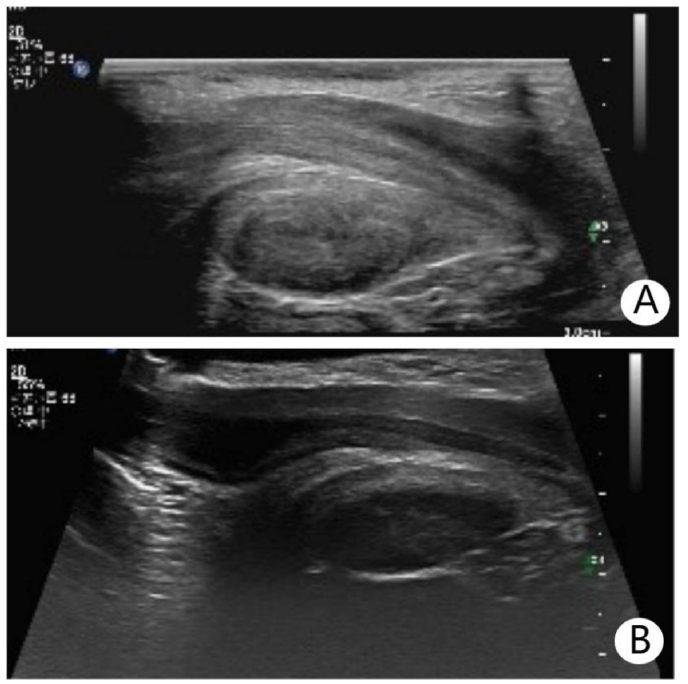
Follow-up color Doppler ultrasound images after completion of anti-inflammatory treatment. The imaging shows the urethra with a normal course, and no significant abnormal mass echoes are observed in the surrounding area.

On physical examination of the penile root, no mass was palpable, and the child did not report pain or discomfort during palpation. After catheter removal, the child experienced smooth urination without dysuria or hematuria and was discharged. Follow-up visits several months post-discharge showed no recurrence of tenderness or urinary symptoms. A repeat ultrasound showed no abnormalities(Fig. 2).

## 3 Discussion

Pediatric urethral masses are an extremely rare lesion, and the etiology remains unclear. After reviewing relevant studies, we found reports mainly on diseases such as rhabdomyosarcoma, urethral stones, fibroepithelial polyps, inflammatory myofibroblastic tumors, urothelial papillomas, neurofibromas, and leiomyomas, with the lesions primarily occurring in the bladder. Urethral masses commonly cause urinary pain or even urinary retention in affected children, making timely catheterization and prevention of acute renal injury particularly important.^4^

Differential diagnosis of urethral masses is crucial. In almost all previous reports on pediatric urethral masses, diagnosis was confirmed through histopathological examination after surgical resection, as preoperative differentiation is highly challenging. In the present case, rhabdomyosarcoma was initially suspected, which is most common in children aged 2–5 years, with the majority located in the anterior lower wall or trigone of the bladder. Ultrasound typically shows a solid mass with low, medium, or high echogenicity and may be associated with a cystic component, often presenting in a grape-like, lobulated, or coral-like pattern.^1^ The ultrasound findings in this case were consistent with an oval cystic mass, with clear borders, poor internal echogenicity, and a layered appearance, showing dense punctate echoes.

This case also warrants differentiation from eosinophilic cystitis, a condition caused by increased eosinophils triggered by celiac disease. The median onset age is approximately 6.5 years, and the main clinical features include elevated absolute and relative eosinophil counts in peripheral blood and bladder wall thickening on ultrasound. In contrast, the peripheral blood analysis in our case showed no eosinophil elevation, though a significant increase in white blood cells was noted.^4^ Fibroepithelial polyps are another benign lesion that can occur in the urethra, with an average onset age of 9 years, predominantly affecting boys. These lesions are usually solitary and smaller than 5 cm in diameter. Urothelial tumors of the bladder are common in adults but rare in children, with the most frequent initial symptom being gross hematuria. Irritative urinary symptoms, such as dysuria, frequency, and urgency, are less common. Ultrasound is the preferred imaging modality, with reported sensitivity ranging from 85 % to 100 %, while CT and MRI may help determine the malignant potential of the lesion^3^.At the same time, it is necessary to distinguish from abscesses. Subcutaneous abscesses generally present with symptoms such as swelling, pain and tenderness, elevated skin temperature and redness, and accumulation of white or yellow pus under the skin. Although abscesses in deep tissues may not have typical symptoms, the possibility of abscess is relatively small based on our strict physical examination. Through literature search, we found that there are currently no reported cases of pediatric urethral abscess, and almost all adult urethral abscess cases have a clear history of gonorrhea, previous periurethral abscess or urethral stricture disease.

All known reports on the treatment of urethral masses have involved transurethral resection of the mass under cystoscopy, followed by histopathological examination for definitive diagnosis.^5^^,^^6^ However, in this case, surgery was not performed, and the child recovered through conservative treatment. Therefore, we believe that not all children with urethral masses require surgery. In particular, when peripheral blood white blood cells, neutrophils, or C-reactive protein are significantly elevated, and ultrasound shows small cystic low-density foci, inflammation should be considered.^7^^,^^8^ In such cases, necessary anti-inflammatory treatment can be administered, with regular follow-up ultrasounds. Spontaneous resolution is possible, and conservative treatment can avoid the trauma to the urethra caused by cystoscopic surgery, while offering faster recovery, less pain, and lower hospitalization costs.^9^, ^10^, ^11^, ^12^ Of course, in children with urinary retention, timely catheterization is critical.

## 4 Conclusions

Urethral masses are a rare but important pediatric urological condition. Most urethral masses cause dysuria, urinary retention, and even acute kidney injury, and due to their atypical imaging features, they are often difficult to accurately diagnose without surgery. Based on the characteristics of this case, we suggest that in cases with significant peripheral blood inflammatory markers and cystic low-density echoes on imaging, anti-infection treatment should be prioritized, which may help avoid surgical intervention.

## CRediT authorship contribution statement

**Hongjia He:** Writing – review & editing, Writing – original draft. **Meng Gui:** Project administration, Methodology. **Kaisheng Li:** Writing – review & editing.
